# Current utility of the ankle-brachial index (ABI) in general practice: implications for its use in cardiovascular disease screening

**DOI:** 10.1186/1471-2296-15-69

**Published:** 2014-04-17

**Authors:** Jane H Davies, Joyce Kenkre, E Mark Williams

**Affiliations:** 1 Faculty of Life Sciences and Education, University of South Wales, Pontypridd CF37 1DL, UK

**Keywords:** Peripheral arterial disease, Doppler ultrasound, Atherosclerosis, Secondary prevention

## Abstract

**Background:**

Peripheral arterial disease (PAD) is a marker of systemic atherosclerosis and associated with a three to six fold increased risk of death from cardiovascular causes. Furthermore, it is typically asymptomatic and under-diagnosed; this has resulted in escalating calls for the instigation of Primary Care PAD screening via Ankle Brachial Index (ABI) measurement. However, there is limited evidence regarding the feasibility of this and if the requisite core skills and knowledge for such a task already exist within primary care. This study aimed to determine the current utility of ABI measurement in general practices across Wales, with consideration of the implications for its use as a cardiovascular risk screening tool.

**Method:**

A self-reporting questionnaire was distributed to all 478 General Practices within Wales, sent via their responsible Health Boards.

**Results:**

The survey response rate was 20%. ABI measurement is primarily performed by nurses (93%) for the purpose of wound management (90%). It is infrequently (73% < 4 times per month) and often incorrectly used (42% out of compliance with current ABI guidance). Only 52% of general practitioners and 16% of nurses reported that patients with an ABI of ≤ 0.9 require aggressive cardiovascular disease risk factor modification (as recommended by current national and international guidelines).

**Conclusion:**

ABI measurement is an under-utilised and often incorrectly performed procedure in the surveyed general practices. Prior to its potential adoption as a formalised screening tool for cardiovascular disease, there is a need for a robust training programme with standardised methodology in order to optimise accuracy and consistency of results. The significance of a diagnosis of PAD, in terms of associated increased cardiovascular risk and the necessary risk factor modification, needs to be highlighted.

## Background

Peripheral arterial disease (PAD) is a marker of systemic atherosclerosis and has been associated with a three to six fold increased risk of death from cardiovascular (CV) causes in multiple longitudinal studies [1]. Moreover, existing evidence demonstrates that PAD (both asymptomatic and symptomatic) conveys independent increased risk in addition to that expected by concomitant traditional CV risk factors and disease [2]. However, PAD is typically asymptomatic and under-diagnosed [3]. This has resulted in calls for the instigation of Primary Care PAD screening which would identify those at increased risk and potentially allow alteration of the disease trajectory via secondary risk factor modification [4]. Current guidelines recommend the same strategy of cardiovascular risk management for persons with PAD as for those with coronary artery disease (CAD) [3,5].

PAD can be diagnosed and also quantified by means of the ankle brachial index (ABI) which involves a comparison of the systolic pressure at the ankle with the systolic pressure at the arm; an ABI of ≤0.9 is considered diagnostic of the disease. The ABI is widely regarded as non-invasive, inexpensive, and easily used in a general practice setting. However, there is limited evidence regarding the feasibility of PAD screening and if the requisite core skills and knowledge for such a task already exist within primary care. Bendermacher et al. considered the workload of screening all patients over the age of 50 in general practices in the Netherlands; they concluded that it was not achievable and suggested a clinical prediction model to determine who should undergo ABI measurement [6]. The Scottish Intercollegiate Guidelines Network (SIGN) state that there is a pool of expertise for measuring the ABI of patients in the community but they do not substantiate this and existing research regarding this issue has produced varying results [7].

This study aimed to determine the current utility of ABI measurement in general Practices across Wales, including: (i) the occupations of those who perform ABI measurement, (ii) frequency of ABI measurement, (iii) reasons for ABI measurement, (iv) methodology utilised for ABI measurement, (v) prior training for ABI measurement and, (vi) subsequent management of patients found to have PAD.

## Method

A self-reporting questionnaire was distributed, via seven health boards, to all general practices within Wales (n = 478); branch practices were not included as staff may work at both main and branch practices which may have resulted in duplication of results. Questionnaires were sent to practice managers and an accompanying letter requested that the survey be passed on to an appropriate person for completion.

Guidelines for the measurement and calculation of the ABI are available from multiple sources [3-5,7-10]. Whilst some are more explicit than others, they all broadly advocate the same methodology (Table 1). The questionnaire (Additional file 1) was designed by the authors to assess six fundamental points of the guidelines advocated ABI method (detailed in Table 2 along with their associated rationales). The questionnaire was piloted at a local general practice and approved by an independent expert (a Consultant Vascular Surgeon) prior to distribution. It is acknowledged that measurement of the ABI includes more complex components such as the choice of Doppler probe frequency and angulation of Doppler probes to achieve good signals; however, the aim of the survey was to determine if the fundamental underpinnings of correct ABI measurement exist.

**Table 1:** Summary of guidelines for the measurement of the Ankle Brachial Index

	**Rest period**	**Equipment for measurement of brachial systolic pressure**	**Number of brachial pulses to be assessed**	**Equipment for measurement of ankle systolic pressure**	**Ankle pulses which should be assessed**	**Method of calculation of the ABI**
**American College of Cardiology/American Heart Association (ACC/AHA) 2005**	Rest supine for 10 minutes	Handheld Doppler ultrasound device & sphygmomanometer	2	Handheld Doppler ultrasound device & sphygmomanometer	Dorsalis Pedis artery and Posterior Tibial artery.	Higher ankle systolic pressure (for that leg) divided by higher brachial pressure of the two arms.
**Scottish Intercollegiate Guidelines Network (SIGN) 2006**	Not mentioned	Handheld Doppler ultrasound device & sphygmomanometer	2	Handheld Doppler ultrasound device & sphygmomanometer	Dorsalis Pedis artery/ Anterior Tibial artery & Posterior Tibial artery. If these cannot be located, assess the Peroneal Artery	Higher ankle systolic pressure (for that leg) divided by higher brachial pressure of the two arms.
**Trans-Atlantic Intersociety Consensus (TASC) 2007**	Not mentioned	Doppler Instrument & sphygmomanometer	2	Doppler Instrument & sphygmomanometer	Dorsalis Pedis artery & Posterior Tibial artery.	Divide both ankle pressures by higher brachial pressures.
**Society for Vascular Technology of Great Britain and Ireland (SVT) 2010**	Rest supine for 5-10 minutes prior to procedure	Handheld continuous wave Doppler ultrasound device & sphygmomanometer	2	Handheld continuous wave Doppler ultrasound device & sphygmomanometer	Dorsalis Pedis artery & Posterior Tibial artery.	Higher ankle systolic pressure (for that leg) divided by higher brachial pressure of the two arms.
**European Society of Cardiology (ESC) 2011**	Not mentioned	Handheld Doppler ultrasound device & sphygmomanometer	2	Handheld Doppler ultrasound device & sphygmomanometer	Posterior Tibial artery & Anterior Tibial artery.	Higher ankle systolic pressure (for that leg) divided by higher brachial pressure of the two arms.
**National Institute for Clinical Excellence (NICE) 2012**	Rest supine when possible. Rest period should be “long enough for blood pressure to return to normal”	Handheld Doppler ultrasound device & sphygmomanometer	2	Handheld Doppler ultrasound device & sphygmomanometer	Three arteries, one of which must be the Peroneal artery as this “may be the only one present in some people, particularly those with diabetes”.	Higher ankle systolic pressure (for that leg) divided by higher brachial pressure of the two arms.
**American Heart Association (AHA)–scientific statement 2012**	Rest 5-10 minutes in supine position	Handheld Doppler ultrasound device & sphygmomanometer	2	Handheld Doppler ultrasound device & sphygmomanometer	Dorsalis Pedis artery & Posterior Tibial artery.	Higher ankle systolic pressure (for that leg) divided by higher brachial pressure of the two arms.

**Table 2:** Aspects of ABI measurement assessed by survey

**Aspect of ABI measurement assessed**	**Recommended by**	**Rationale**
1. Patient rested in supine position for at least 10 minutes prior to ABI measurement?	SVT [9]	• ABI averages 0.35 higher in the seated position as opposed to supine [10].
	NICE [5]	• There is no evidence to recommend a minimum period but it should be long enough for blood pressure to return to normal [5]. The effect of the duration of the rest period on the reliability of the ABI measurement is unknown, with most studies using 5-10 minutes [5].
	AHA [10]	
2. Equipment needed to measure the brachial systolic blood pressure correctly identified as being a Doppler Ultrasound and sphygmomanometer	All guidelines [3-5,7-10]	• Using the Korotkoff method to measure the brachial pressure has been shown to yield lower values compared to Doppler [11].
		• Similarly, automated oscillometric blood pressure devices have been shown to underestimate brachial pressure [12,13].
		• As the brachial pressure forms the denominator of the ABI, underestimation will result in falsely elevated ABIs.
3. Brachial systolic pressure measured in both arms	All guidelines [3-5,7-10]	• A pressure difference between left and right brachial arteries of at least 20 mmHg is present in 3.5% of normal healthy population [14].
		• A recent meta-analysis found that a difference of 15 mmHg or more is actually associated with 2.5 times increased risk of PAD [15].
		• It is therefore paramount that both brachial pressures are measured to prevent missed diagnoses and/or in correct classification of PAD.
4. Equipment needed to measure the ankle systolic blood pressure correctly identified as being a Doppler Ultrasound and sphygmomanometer	All guidelines [3-5,7-10]	• Oscillometric devices have been found to overestimate ankle systolic pressure [16] resulting in falsely elevated ABIs and reduced sensitivity for detecting PAD [17-19].
		• Most oscillometric devices are unable to detect low pressures (<50 mmHg) and hence recording failures are frequent in cases of moderate to severe PAD [10].
5. More than one pulse assessed at each ankle/foot	All guidelines [3-5,7-10]	• Guidelines differ with regard to which of the three ankle arteries should be assessed, although they all agree that it should be more than one.
		• NICE guidance specifies that the arteries assessed should always include the peroneal artery as this may be the only one present in some people, particularly those who are diabetics [5].
6. ABI calculated by dividing the higher of the ankle systolic blood pressures by the higher of the brachial systolic blood pressures	All guidelines [3-5,7-10]	• Although several authors have argued that utilising the lower ankle systolic pressure as the numerator in the ABI would result in greater sensitivity for the identification of early PAD [20,21], others have argued that the higher pressure should be used to prevent over diagnosis in healthy subjects [10].
		• Others argue that standardisation of the calculation is the important issue, because this would optimise accuracy and consistency of results universally hence ensuring PAD diagnoses are based on the same parameters [22,23].

As general practice survey response rates are often low [24], several strategies were employed in an attempt to address this issue: the questionnaire was designed to be minimally time consuming with predominantly close-ended, tick box questions, with a pre-paid return envelope included. Returned questionnaires were entered into a prize draw (a £50 gift voucher for each heath board).

This study did not require ethical approval (according to the UK Health Research Authority guidance). However, approval to distribute the questionnaire was obtained from each of the individual health boards and completion of the survey constituted consent.

## Results

The overall response rate was 20% (95:478) and ranged from 16-41% across individual health boards: Cwm Taf Health Board 16% (8:50), Aneurin Bevan Health Board 22% (20:91), Cardiff & Vale University Health Board 19% (13:68), Abertawe Bro Morgannwg Health Board 18% (14:77), Betsi Cadwaladr University Health Board 16% (19:119), Powys Teaching Health Board 41% (7:17), Hywel Dda Health Board 25% (14:56). Thirty per cent (27:95) of returned surveys were completed by GPs, 6% (6:95) by nurse practitioners, 34% (32:95) by practice nurses and 5% (5:95) by district nurses. The remaining 25% were completed as a collaboration between GPs and nursing staff.

Twenty seven per cent (26:95) of responding general practices were not undertaking ABI measurement, with patients needing this procedure often being referred to secondary care. Other practices relied on their district nursing colleagues (who, in Wales, are not generally based within general practices) to undertake the task.

**Figure 1:**
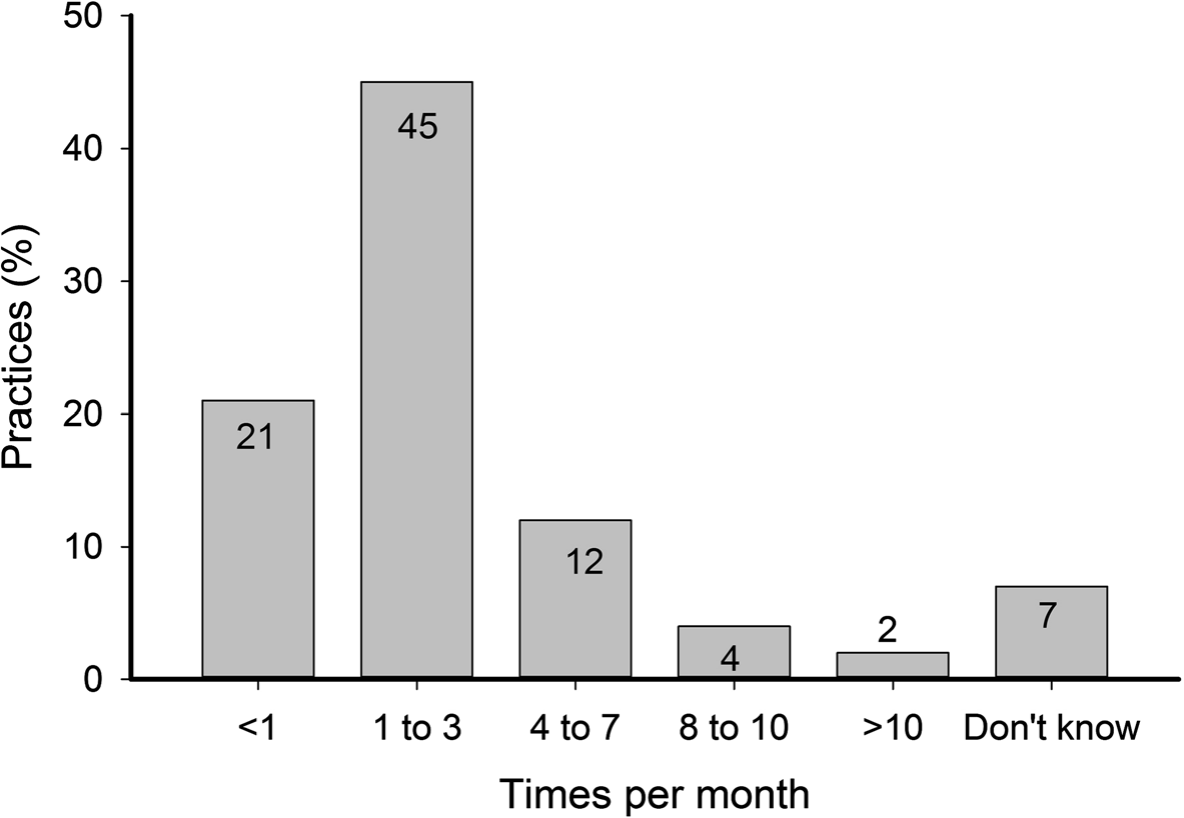
Frequency of ABI measurement within general practices.

**Figure 2:**
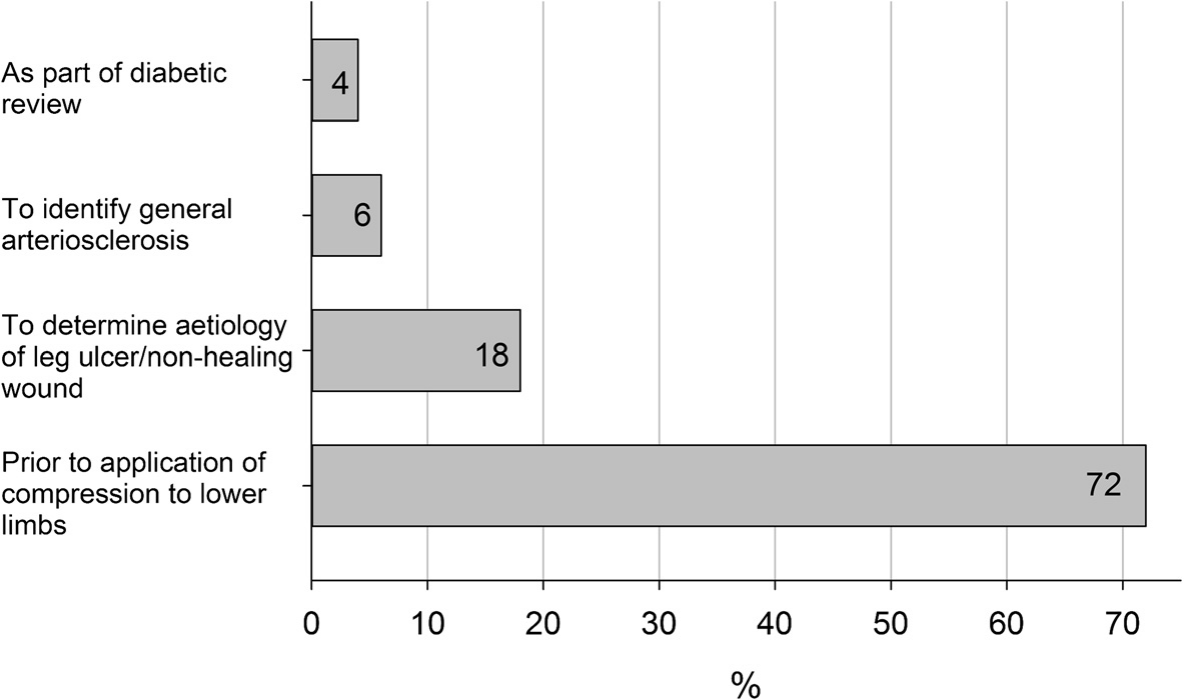
Reasons for ABI measurement.

The majority of practices reported performing ABI measurements relatively infrequently at less than four times a month (73%) (Figure 1). Respondents were asked to indicate if there were any other reasons, besides the presence of signs and symptoms of lower limb arterial insufficiency, which would cause them to undertake or request ABI measurement. Whilst the management of lower limb oedema and leg ulceration/wounds accounted for 90% of responses to this question, it was interesting to note that 6% reported utilising the ABI in a screening capacity (Figure 2).

General practitioners (GPs) were the least likely occupational group to undertake ABI measurement. They were also the least likely to: (i) consider themselves, or be considered by colleagues, to be competent at ABI measurement, (ii) have received formal training for ABI measurement, and (iii) be compliant with current guidelines for ABI measurement (Table 3). Conversely, practice nurses were the most likely to perform ABI measurement with 64% having received training for the procedure and 71% of practice nurse survey responders being compliant with ABI measurement guidelines. In general, nurses were much more likely to have received training for ABI measurement and more likely to be adhering to current ABI guidelines.

**Table 3:** Summary of survey results

	**General practitioners**	**Practice nurses**	**Nurse practitioners**	**District nurses**	**Overall**
% Who typically performs ABI measurement within General Practices?	5.2 (5/95)	50.5 (48/95)	7.4 (7/95)	9.5 (9/95)	72.6%
					[remaining 27.3% referred to secondary care (15.8%) or DN teams not based within General Practices (11.6%)]
% who consider themselves or are considered by colleagues to be competent at ABI measurements	11	48	56	60	32
*Training*					
• % of General Practices with staff trained for ABI measurement	3	30	4	5	42
• % of respondents who currently undertake ABI measurement *and* have received ABI training	20	64	43	100	65
*ABI Methodology*					
% who correctly identified ABI method and equipment according to current guidelines:					
• All respondents	38	71	80	100	61
• Respondents currently undertaking ABI measurement	0	68	80	100	66
(breakdown of individual assessment points below)					
1. % who would rest patients prior to ABI measurement					
• All respondents	65	93	100	100	82
• Respondents currently undertaking ABI measurement	0	89	100	100	81
					[reasons for not resting patients: lack of time 76% (13:17); not considered necessary 24% (4:17)]
2. % who identified correct equipment used for Brachial SBP measurement					
• All respondents	80	93	80	100	87
• Respondents currently undertaking ABI measurement	80	96	100	100	95
3. % who said they would measure the brachial SBP in both arms					
• All respondents	86	93	100	100	87
• Respondents currently undertaking ABI measurement	20	93	100	100	86
4. % who identified correct equipment used for Ankle SBP measurement					
• All respondents	88	96	100	100	93
• Respondents currently undertaking ABI measurement	80	96	100	100	86
5. % who said they would assess more than one foot/ankle arteries					
• All respondents	83	93	100	100	90
• Respondents currently undertaking ABI measurements	60	93	100	100	91
6. % who said they would calculate ABI by dividing the highest ankle SBP by the higher brachial SBP					
• All respondents	46	75	100	100	67
• Respondents currently undertaking ABI measurements	20	79	100	100	77
				
% who experience difficulty locating ankle/foot pulses	54	59	40	100	59
% who experience difficulty maintaining position of Doppler probe whilst simultaneously pumping up BP cuff	39	33	20	20	33

**Figure 3:**
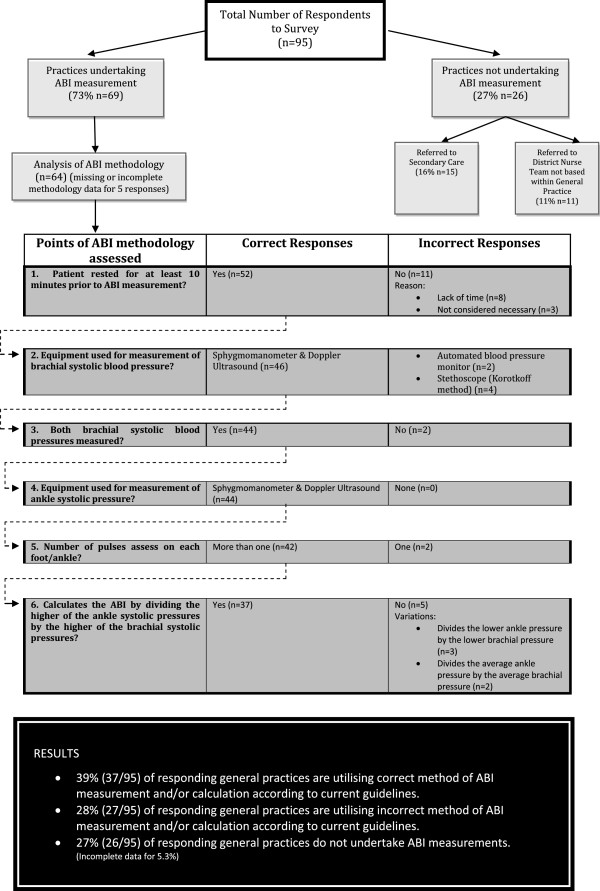
Diagrammatic representation of survey responses.

There was considerable variation in the method utilised for ABI measurement and calculation. Only 58% of general practices undertaking ABI measurements were found to be compliant with current guidelines for the procedure. Figure 3 illustrates the proportion of survey responses which successfully progressed through each of the methodology assessment points as described in Table 2. Eighteen per cent of practices reported not resting their patients in the supine position prior to ABI measurement. Lack of time was the primary reason for not doing this (75%), whilst the remaining 25% of respondents thought it was unnecessary. Five per cent of respondents reported utilising the Korotkoff method to measure the brachial systolic pressure with a further 2% reportedly using automated blood pressures devices. Furthermore, 13% of respondents said that they would measure the brachial systolic pressure in one arm only. Thirty three per cent of respondents reported not calculating ABI’s according to current guidance. In 17% of cases, this was because only one brachial pressure and/or only one ankle pressure had been measured. A further 12% reported using the lower of the ankle and/or brachial pressures, whilst the remaining 4% used the average of the ankle and/or brachial pressures when calculating the index.

A large proportion of respondents reported difficulty in (i) locating pulses in the foot/ankle (59%), and (ii) maintaining the position of the Doppler probe whilst inflating the blood pressure cuff (33%). The survey provided opportunity to expand on these issues and 9% of respondents (all of which were nurses) independently stated that they addressed these problems by utilising another health professional to assist with the procedure.

Seventy six per cent (28:37) of respondents who were in compliance with current guidelines for ABI measurement reported having received formal training for the procedure. Accordingly, 73% (38:52) of respondents who were not in compliance with current guidelines had not received any formal training.

**Figure 4:**
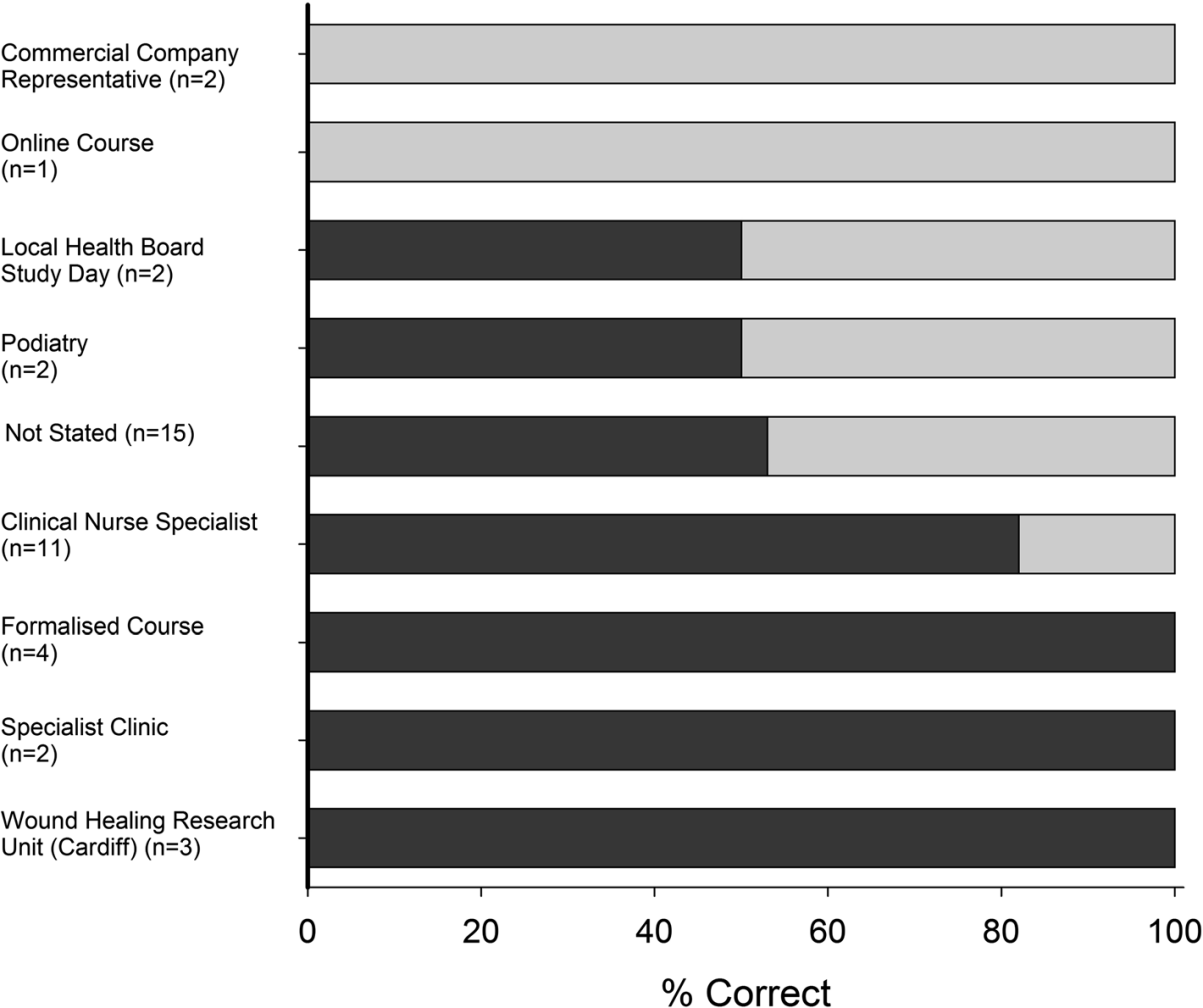
**Correct ABI measurement according to origin of training.** Clinical Nurse Specialist = Tissue Viability Nurse/Wound Care Practitioner, Specialised Clinic = Local leg ulcer clinic/lymphoedema clinic, Formalised Course = Wound Management Course/Diabetic Diploma.

Training originated from a variety of sources with Tissue Viability Nurses/Wound Care Practitioners accounting for the largest proportion (41%). Eighty two per cent of respondents who received training from these clinical nurse specialists reporting measuring ABI’s in accordance with current guidance. Training via specialist clinics or as part of a formalised course also appears effective in achieving compliance with guidelines (Figure 4). Five per cent of respondents expressed their frustration at a lack of refresher or update ABI education/courses to enable them to maintain their competency in the procedure.

Respondents were asked to indicate any medical management which they would instigate or expect to be instigated for patients who were found to have PAD. Twenty nine per cent referred to “aggressive” cardiovascular risk factor modification such as commencing antiplatelets, control of hypertension and hyperlipidaemia in combination with lifestyle advice; this is in line with current guidance issued by the European Society of Cardiology (ESC) and National Institute of Clinical Excellence (NICE) [3,5]. A further 8% mentioned a lesser degree of cardiovascular risk modification involving only lifestyle factors such as encouraging smoking cessation and exercise. GPs were more likely to have mentioned cardiovascular disease risk factor modification than nurses (56% versus 16%).

## Discussion

Results indicate that ABI measurement is very much a nursing task which is, at present, mainly performed for the purpose of wound management rather than for cardiovascular risk assessment. It is only utilised at approximately three quarters of respondents from general practices in Wales and those that do utilise it, do so on an infrequent basis. According to a literature review conducted by Sihlangu & Bliss [25], this raises issues of competency as studies have demonstrated greater variability in ABI’s when measured by less experienced practitioners [26,27]. In addition, this survey found that a large proportion of respondents experienced difficulties with the skilled or technical aspects of the procedure such as locating ankle pulses and maintaining the position of the Doppler probe, and it is possible that these difficulties were also related to inexperience. A survey by Mohler et al. found that primary care staff reported increased use of the ABI following their participation in a PAD/ABI training programme [28]. However, the survey was completed 1-3 months following programme completion so it is not known if this increase would have been sustained over a longer time period. This survey found that reported use of the ABI was low regardless of whether training had been received or not.

Aboyans and colleagues recently highlighted that a lack of standardised ABI methodology is likely to have significant clinical, public health and economic repercussions [10]. They subsequently released a scientific statement setting out an evidence based, recommended procedure for ABI measurement and interpretation [10]; this concurs with the methodology assessed by this survey. The clinical rationale for standardisation arises from the fact that the majority of studies demonstrating the association between low ABI and CV risk, have used this recommended methodology and it is not known if this would differ with alternative methods.

This survey has found that deviations from the guideline advocated method of ABI measurement are commonplace and two inter-related factors have emerged as important with regard to this. The first concerns the time it takes to perform the measurement, as the majority of deviations could be attributed to attempts to reduce this. Not resting patients prior to measurement, using automated blood pressure monitors, measuring the brachial pressure in one arm only and assessing only one ankle pulse all equate to a reduction in the time it takes to perform the test. Mohler et al. [28] and Bendermacher [6] found that lack of time was a barrier to the use of the ABI in primary care. This issue is further compounded by the fact that the procedure sometimes requires two health care personnel. Results indicate that GPs are more likely to resort to these time saving strategies and this is not surprising considering that their allocated time for a complete patient consultation is often only 10 minutes.

The second factor concerns training, with those who had undergone specialised training for the procedure being much more likely to be adhering to the guidelines advocated method. Hence, it appears that training successfully educates practitioners regarding the importance of not “cutting corners” at the expense of the accuracy of results. Mohler et al. found that a targeted educational initiative can have significant impact on the use of the ABI in clinical practice which could offer dramatic benefits to improve PAD diagnosis [28].

### Management of PAD patients

The under treatment of PAD patients has been well documented; the global Reach Registry demonstrated that patients with PAD were significantly less likely to be at target blood pressure, cholesterol and glucose levels in comparison to patients with coronary artery disease or cerebrovascular disease [21]. The recent publication of PAD guidelines by various organisations [3,5] and the addition of PAD indicators to the 2012/13 Quality and Outcomes Framework [29] in the UK may have served to increase awareness and improve the treatment of PAD. In addition, general practice computer software systems in the UK, such as EMIS (Egton Medical Information Systems), now generate pop-up reminders to consider aspirin, check BP and cholesterol when coding a new diagnosis of peripheral arterial disease. It is difficult to establish if data from this survey represent improved medical management of PAD patients. It is clear however, that the large majority of nurses who responded to the survey consider the ABI only in terms of its repercussions for leg ulcer/wound management and are unaware of its association with increased cardiovascular risk.

### How this fits in

The global perspective of PAD screening is far from definitive; it is not universally advocated across international guidelines, and there is no consensus regarding who should be targeted. According to the United States Preventive Services Task Force [30] there is insufficient evidence to recommend PAD screening and this is based on a lack of randomised control trials of PAD screening versus no screening. Additionally, whilst some countries now offer remuneration for ABI measurement in Primary Care (e.g. the Netherlands, Australia), this is not the case in the UK or USA.

This survey has identified that a further potential issue of PAD screening relates to ABI measurement as the recommended screening tool. Its underutilisation and often incorrect use within general practice appears to be related to lack of time, but also suggests a current knowledge and skills deficit.

### Study strengths and limitations

The response rate was low but not atypical, as published medical practitioners response rates are often lower than 30% [31,32]. Mohler et al. utilised a survey to assess the utility and barriers to the use of the ABI in primary care practice. Primary care staff (physician and non-physicians) that had one month previously undergone a PAD and ABI preceptorship programme were either given or mailed the survey. It could be assumed that this participation in an educational programme would have served to raise awareness of the relevance of the survey and yet the response rate was still only 24% [28]. Nevertheless, the possibility of response bias needs to be borne in mind when considering results of this survey. It is possible that those who do not utilise the ABI may have been less likely to complete the survey and hence its use may be over-estimated. Furthermore, the small number of nurse practititoner and district nurse respondents means that results relating to these occupational groups may be less representative of the professions as a whole. These limitations acknowledged, this survey is, to the authors’ knowledge, the only assessment of the utility of the ABI in the UK. Representation from both nurses and physicians from general practices in all areas of Wales has been achieved.

This study targeted primary care practitioners that were based within general practices as it is here that screening strategies are likely to be undertaken. It is acknowledged that ABI skills and knowledge exist in other sectors of primary care such as district nursing teams and podiatry for example. In addition, the usual validity concerns regarding self-reported behaviour in surveys apply and issues such the accuracy and reproducibility of ABI measurements have not been addressed. Hence these two points provide a focus for future research.

## Conclusions

ABI measurement is an under-utilised and often incorrectly performed procedure in the surveyed general practices; lack of time and inadequate training have been identified as factors associated with this finding. Previous research undertaken in the USA [28] and the Netherlands [33] made remarkably similar conclusions hence demonstrating that these identified issues are historically problematic and not confined to Wales and the UK.

Prior to the potential adoption of the ABI as a formalised screening tool for cardiovascular disease, there is a need for a robust training programme with standardised methodology in order to optimise accuracy and consistency of results. ABI Training programmes should include the methodological requirements for accurate and reproducible ABI measurement, as well as the theoretical basis and limitations of the test. The subsequent implications of a reduced ABI with regard to cardiovascular risk also need to be highlighted.

## Abbreviations

ABI: Ankle brachial Index; CAD: Coronary artery disease; CV: Cardiovascular; PAD: Peripheral arterial disease; GP: General practitioner; EMIS: Egton Medical Information Systems Limited.

## Competing interests

All authors declare that they have no competing interests.

## Authors’ contributions

JHD and EMW designed the study. JK assisted in obtaining permission from Health Boards to distribute the questionnaire. JHD distributed questionnaires, coded responses where necessary and collated results. JHD, JK and EMW interpreted results. JHD and EMW wrote the paper. All authors read and approved the final manuscript.

## Authors’ information

This study was undertaken as part of JHD’s PhD which is being undertaken at the University of South Wales. EMW is JHD’s director of studies. JK is Professor of Primary Care at the University of South Wales and is also part of JHD’s supervisory team.

## Pre-publication history

The pre-publication history for this paper can be accessed here:

http://www.biomedcentral.com/1471-2296/15/69/prepub

## Supplementary Material

Additional file 1 Survey: Use of the Ankle Brachial Index in General Practice. Click here for file
