# Supplementary material for: Current utility of the ankle-brachial index (ABI) in general practice: implications for its use in cardiovascular disease screening
Source: BMC Fam Pract. 2014 Apr 17;15:69. doi: 10.1186/1471-2296-15-69 (PMC4021160; doi:10.1186/1471-2296-15-69)
Supplement: Additional file 1 — Survey: Use of the Ankle Brachial Index in General Practice. [file 1471-2296-15-69-S1.docx]

1. Please tick the occupation of the person completing this survey: GP

Nurse Practitioner

Practice Nurse

Other

(please specify) ...................................

**Reasons for Ankle Brachial Index (ABI) Measurement**

1. If you suspect a patient to have Peripheral Arterial Disease, how would you confirm this? (Please tick all that apply)
   1. Clinical Examination
   2. Edinburgh Claudication Questionnaire

- 1. ABI measurement
  2. Other (please state)

....................................................................................................................................................

1. Besides signs and symptoms of arterial insufficiency, are there any other reasons which would cause you to perform or request ABI measurement? If yes, please state:

YES NO

(If ‘YES’ please state) ...............................................................................................................................

**ABI Measurement**

1. If you wanted to determine a patient’s ABI, would you carry out the measurement procedure yourself?

YES NO

If you answered ‘NO’ to question 4, please indicate the occupation of the person who would carry out the measurement (please tick):

- 1. GP
  2. Nurse Practitioner
  3. Practice Nurse
  4. District Nurse
  5. Other

(please specify) .............................................

1. Please estimate how often, in an average month, ABI measurements are done in your practice?
   1. 0 times
   2. 1-3 times
   3. 4-7 times
   4. 8-10 times
   5. >10 times
   6. Don’t know
2. a. Please state the total number of GP’s at your practice:
3. How many of these are competent at ABI measurement:
4. a. Please state the total number of Nurse Practitioners at your practice:

b. How many of these are competent at ABI measurement:

1. a. Please state the total number of Practice Nurses at your practice:

b. How many of these are competent at ABI measurement:

1. a. Please state the total number of District Nurses at your practice:

b. How many of these are competent at ABI measurement:

1. Has anyone in your practice received specialist training for ABI measurement?

YES NO

If yes, please state who has received training and from whom: ............................................................................

................................................................................................................................................................................

.................................................................................................................................................................................

1. Please answer the following questions regarding the procedure of ABI measurement.
   1. Do you rest your patient in a supine position for at least 10 minutes prior to undertaking the procedure? YES No

If you answered No to the above, please tick reason why:

Don’t think it is necessary

Lack of time

Other (please state) Reason:......................................................................................

- 1. Sometimes do you find the procedure of ABI Measurement difficult because of:

|  | YES | NO |
| --- | --- | --- |
| Difficulty finding pulses? |  |  |
| Difficulty holding the Doppler Probe in place whilst pumping up  the blood pressure cuff? |  |  |
| Difficulty in the actual calculation of the ABI? |  |  |

Please state any other difficulties you have experienced with ABI measurement:

....................................................................................................................................................................

......................................................................................................................................................

.................................................................................................................................................

- 1. When measuring the *brachial* pressure, please indicate all of the pieces of equipment which you would use (please tick)
     1. Automatic blood pressure monitor
     2. Manual blood pressure sphygmomanometer
     3. Stethoscope
     4. Doppler probe
     5. Other

(Please state)........................................................................................................................................

- 1. When measuring the *brachial* pressure, do you:
     1. Measure the BP in 1 arm only
     2. Measure the BP in both arms
  2. When measuring the *ankle* pressures, what equipment would you use?
     1. Automatic blood pressure monitor
     2. Manual blood pressure sphygmomanometer
     3. Stethoscope
     4. Doppler probe
     5. Other

(Please state)........................................................................................................................................

- 1. When measuring the ankle pressures, what pulses would you assess? (Please tick all that apply)
     1. Dorsalis pedis
     2. Posterior tibial
     3. Anterior tibial
     4. Peroneal
  2. If you measured the brachial pressure in both arms, when calculating the result do you use the:
     1. Higher of the brachial pressures
     2. Lower of the brachial pressures
     3. Average of the brachial pressures
     4. N/A because only 1 measurement made
  3. If you measured more than 1 ankle pressure, when calculating the result do you use the:
     1. Higher of the ankle pressures
     2. Lower of the ankle pressures
     3. Average of the ankle pressures
     4. N/A because only 1 measurement made

**Referral to Secondary Care**

1. Would you refer a patient for a vascular opinion without having done an ABI measurement?

YES NO

1. Is there an ABI cut off point at below which you would always refer to secondary care for a vascular opinion? For example, would you refer all patients with an ABI of 0.9 or less for a vascular opinion?

YES NO

If yes, please state the cut off ABI value: .....................

Please expand on your answer if you wish:.................................................................................................

............................................................................................................................................................

1. If a patient showed signs or symptoms of PAD or had an ABI of less than 0.9 and you chose not to refer for a vascular opinion, please briefly outline your management of the patient.

................................................................................................................................................................

..............................................................................................................................................................

............................................................................................................................................................
